# Effect of the protease plasmin on *C. elegans* hyperactive DEG/ENaC channels MEC-4(d) and UNC-8(d)

**DOI:** 10.17912/micropub.biology.000412

**Published:** 2021-06-21

**Authors:** Christina K. Johnson, David D. Miller, Laura Bianchi

**Affiliations:** 1 University of Miami; 2 Vanderbilt University

## Abstract

*C. elegans* MEC-4 and UNC-8 belong to the DEG/ENaC family of voltage-independent Na^+^ channels and have been implicated in mechanosensation and synaptic remodeling. MEC-4 and UNC-8 hyperactive mutants, designated (d) mutants, conduct enhanced currents and cause cell death due to uncontrolled influx of cations. We show here that MEC-4(d) but not UNC-8(d) currents are further potentiated by treatment with the protease plasmin and that this effect is dependent upon co-expression with the chaperon protein MEC-6. Mammalian DEG/ENaC channels are cleaved by plasmin in the channel finger domain and both MEC-4 and UNC-8 have a predicted plasmin cleavage site in this domain. We previously showed that MEC-4(d), but not UNC-8(d), currents are increased by co-expression with MEC-6, which interacts with the channel via the finger domain. We suggest that interaction of the channel subunit with MEC-6 may render the plasmin cleavage site more accessible. Given that *C. elegans* expresses a homolog of plasmin, these effects might be relevant *in vivo*.

**Figure 1. Plasmin enhances MEC-4(d) but not UNC-8(d) currents in a MEC-6-dependent manner:**
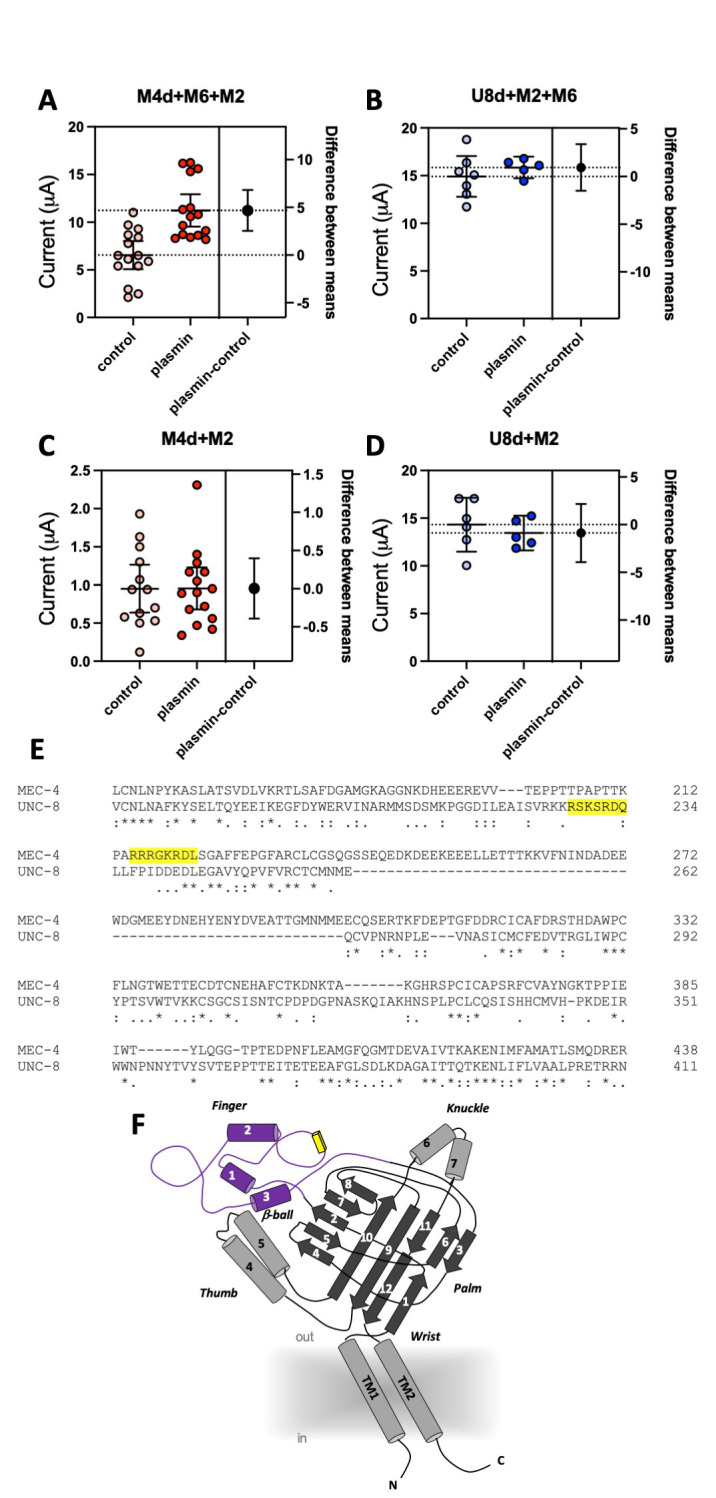
Estimation plots by unpaired t-Test analysis of currents recorded in oocytes not treated and treated with plasmin (Calin-Jageman and Cumming 2019).Individual data points, means and 95% confidence intervals are shown.In each graph,on the right, the black circle and the error bars indicate the difference between the means and its 95% confidence interval. **A:** Currents recorded at -100 mV from oocytes expressing MEC-4(d)+MEC-2+MEC-6 not treated (light red) and treated (dark red) with 10 μg/ml plasmin, as indicated. N = 15, each. The difference between the means was 4.68 (71% increase), the lower and upper 95% confidence levels were 2.45 and 6.83 respectively (38% and 104% change, respectively). **B:** Same as panel A for oocytes expressing UNC-8(d)+MEC-2+MEC-6 not treated (light blue) and treated (dark blue) with plasmin. N = 7 and 5. The difference between the means was 0.94 (6% increase), the lower and upper 95% confidence levels were -1.50 and 3.38 respectively (-10% and 22% change, respectively). **C:** Same as in A for oocytes expressing MEC-4(d)+MEC-2 not treated (light red) and treated (dark red) with plasmin. N = 15 and 13 respectively. The difference between the means was 0.003 (0% change), the lower and upper 95% confidence levels were -0.391 and 0.398 respectively (-41% and 41% change; such large percentage changes are due to the relatively large variability of small currents). **D:** Same as panel A for oocytes expressing UNC-8(d)+MEC-2 not treated (light blue) and treated (dark blue) with plasmin. N = 6 and 5. The difference between the means was -0.87 (6% decrease), the lower and upper 95% confidence levels were -3.92 and 2.17 respectively (-27% and 15% change, respectively). **E:** Alignment of the finger domain of MEC-4 and UNC-8 containing the predicted plasmin sites (yellow background). **F:** Schematic representation of a DEG/ENaC channel subunit based on its crystal structure (Jasti, Furukawa *et al.* 2007). Alpha helixes are shown as cylinders and beta sheets are represented as flat arrows. The finger domain (purple) contains the plasmin recognition site (yellow box). The shaded area around transmembrane domains 1 and 2 (TM1 and TM2) represents plasma membrane.

## Description

MEC-4 and UNC-8 are subunits of the DEG/ENaC family of voltage-independent Na^+^ channels in *C. elegans* (Driscoll and Chalfie 1991, Canessa, Horisberger *et al.* 1993, Waldmann, Champigny *et al.* 1996, Waldmann, Champigny *et al.* 1997, de Weille, Bassilana *et al.* 1998, Waldmann and Lazdunski 1998). While MEC-4 is expressed in body touch neurons where it mediates the transduction of gentle touch sensation (Driscoll and Chalfie 1991, O’Hagan, Chalfie *et al.* 2005), UNC-8 is primarily expressed in motoneurons where it is involved in synaptic remodeling during development (Tavernarakis, Shreffler *et al.* 1997, Miller-Fleming, Petersen *et al.* 2016). Both MEC-4 and UNC-8 can be hyperactivated by genetic mutations that hinder channel closing, called (d) mutations (Driscoll and Chalfie 1991, Shreffler, Magardino *et al.* 1995, Goodman, Ernstrom *et al.* 2002, Wang, Matthewman *et al.* 2013). *C. elegans* neurons and *Xenopus* oocytes expressing these hyperactive variants of MEC-4 and UNC-8 undergo cell death due to uncontrolled flux of ions into the cell. Cell death in *Xenopus* oocytes and in cultured *C. elegans* neurons can be prevented by incubation with the DEG/ENaC channel blocker amiloride (Goodman, Ernstrom *et al.* 2002, Suzuki, Kerr *et al.* 2003, Wang, Matthewman *et al.* 2013).

Potentiation of the mammalian α, γ, and δ subunits of the DEG/ENaC ENaC subfamily can be also achieved via proteolytic cleavage of the extracellular domain (Kleyman and Eaton 2020). This cleavage results in the release of inhibitory peptides and probably the conformational change of the channel that leads to larger whole-cell currents. DEG/ENaC proteolytic processing begins in the Golgi by the Golgi resident protease furin (Hughey, Mueller *et al.* 2003, Hughey, Bruns *et al.* 2004) and is completed once the channel arrives at the cell surface by transmembrane and extracellular proteases such as plasmin (Passero, Mueller *et al.* 2008). While the effect of proteases on mammalian DEG/ENaCs has been extensively studied, little is known about whether nematode DEG/ENaC subunits are sensitive to these enzymes.

We coexpressed MEC-4(d) with stomatin-like protein MEC-2 and paraoxonase-like protein MEC-6, and, in a separate experiment, UNC-8(d) with MEC-2 and MEC-6 in *Xenopus* oocytes and recorded currents using the two-electrode voltage-clamp technique. In the same batch of oocytes, we compared current amplitudes recorded at -100 mV in cells treated with 10 μg/ml of plasmin (in ND96) for 30 minutes with currents recorded in mock-treated cells (ND96). We found that MEC-4(d) currents were nearly doubled in plasmin-treated oocytes (Fig. 1A) (6.54 +/- 0.68 and 11.23 +/- 0.79 for mock and plasmin-treated cells respectively, mean +/- SE, n=15 each, p=0.0001 by unpaired t-Test). However, plasmin did not increase UNC-8(d) current amplitude (Fig. 1B) (14.91 +/- 0.97 and 15.85 +/- 0.40 for mock and plasmin-treated cells respectively, mean +/- SE, n=7 and 5). Mammalian ENaCs are cleaved by plasmin in the channel finger domain where this protease has at least one recognition site (Passero, Mueller *et al.* 2008). We thus used the SitePrediction website (https://www.dmbr.ugent.be/prx/bioit2-public/SitePrediction/) to predict potential plasmin cleavage sites in the finger domains of MEC-4 and UNC-8 (Fig. 1E and F). We found that both proteins contain at least one potential plasmin cleavage site in the finger domain (Fig. 1E, highlighted in yellow). This suggests that lack of effect of plasmin on UNC-8(d) is not due to lack of potential recognition sites for plasmin in UNC-8.

MEC-6 is a paraoxonase-like protein (Chelur, Ernstrom *et al.* 2002) with chaperone function (Chen, Bharill *et al.* 2016) that increases the number of functional MEC-4 channels at the cell surface (Brown, Liao *et al.* 2008, Chen, Bharill *et al.* 2016). We recently showed that MEC-6 mediates MEC-4(d) regulation via interaction with the channel finger domain (Matthewman, Johnson *et al.* 2018). We also showed that MEC-6 does not enhance UNC-8(d) currents in oocytes. We thus postulated that the effect of plasmin on MEC-4(d) might depend on MEC-6 and that the lack of effect on UNC-8 might be related to the fact that MEC-6 does not change UNC-8 currents in *Xenopus* oocytes. To test this hypothesis, we repeated the experiments in oocytes injected with only MEC-4(d) and MEC-2 or with only UNC-8(d) and MEC-2, but without MEC-6. As previously reported, MEC-4(d) currents are much smaller in the absence of MEC-6 (Chelur, Ernstrom *et al.* 2002). More importantly, we found that plasmin does not increase MEC-4(d) currents in oocytes that do not express MEC-6 (Fig. 1C) (0.95 +/- 0.14 and 0.95 +/- 0.13 for mock and plasmin treated cells respectively, mean +/- SE, n=13 and 15). UNC-8(d) currents remain unaffected by plasmin treatment (Fig. 1D) (14.32 +/- 1.09 and 13.44 +/- 0.65 for mock and plasmin treated cells respectively, mean +/- SE, n=6 and 5). Taken together, these results support the idea that chaperon MEC-6 is needed for enhancement of MEC-4(d) current amplitude by protease plasmin. Interestingly, Brown and colleagues showed that activation of MEC-4(d) by extracellular chymotrypsin is independent of both MEC-2 and MEC-6 (Brown, Liao *et al.* 2008). Collectively, these data suggest that *C. elegans* DEG/ENaC channels might be sensitive to different extracellular proteases based, not only on channel primary sequence but also on its quaternary structure. These results also suggest that chaperon MEC-6 may help expose potential cleavage sites. Given that *C. elegans* expresses *svh-1*, the homolog of mammalian plasmin precursor plasminogen, we suggest that these effects might be relevant *in vivo* both in physiological conditions and when DEG/ENaC channels are hyperactivated (Hisamoto, Li *et al.* 2014).

## Methods

**Molecular biology**

Plasmids encoding MEC-4(d) and MEC-2 were a gift from the Chalfie lab (Goodman, Ernstrom *et al.* 2002). The MEC-6 and UNC-8(d) cDNA were cloned in a PGEM vector and were published previously (Bianchi, Gerstbrein *et al.* 2004, Wang, Matthewman *et al.* 2013, Matthewman, Miller-Fleming *et al.* 2016, Matthewman, Johnson *et al.* 2018). cRNAs were synthesized using T7 mMESSAGE mMACHINE kit (Invitrogen) following manufacturer’s protocols. cRNAs were purified, run on denaturing agarose gels for size and integrity verification, and quantified spectroscopically.***Xenopus* oocytes expression and electrophysiology**Stage V-VI defolliculated oocytes from *Xenopus laevis* were purchased from Ecocyte Bioscience US LLC (Austin, Texas) and injected with 10 ng/oocyte of each cRNAs except MEC-6 cRNA, which was injected at 2 ng/oocyte. Injected oocytes were incubated in ND96 medium consisting of 96 mM NaCl, 2 mM KCl, 1.8 mM CaCl_2_, 1 mM MgCl_2_, and 5 mM HEPES, pH 7.5, supplemented with penicillin and streptomycin (0.1 mg/ml) and 2 mM Na-pyruvate at 20°C. The next day the medium was changed to ND96 plus 500 µM amiloride to prevent cell death(Goodman, Ernstrom *et al.* 2002). Whole-cell currents were measured three days after the injection using a two-electrode voltage-clamp amplifier (GeneClamp 500B; Axon Instruments) at room temperature. Electrodes with resistance between 0.3–0.7 MΩ were filled with 3 M KCl. To measure MEC-4(d) currents, oocytes were perfused with a physiological NaCl solution containing (mM): 100 NaCl, 2 KCl, 1 CaCl_2_, 2 MgCl_2_, and 10 HEPES, pH 7.2 (NaOH). Due to the strong block by divalent cations (Wang, Matthewman *et al.* 2013), to measure UNC-8(d) currents a divalent cation–free NaCl solution containing (mM): 110 NaCl, 2 KCl, 1 EGTA, and 10 HEPES, pH 7.2 (NaOH) was used. To elicit the whole-cell currents, the oocyte membrane was clamped at −30 mV and stepped from −160 to +100 mV in 20 mV increments. Current amplitude was measured at -100 mV and 300 ms. We used the pCLAMP suite of programs (Axon Instruments) for data acquisition and analysis. Currents were filtered at 200 Hz and sampled at 1 kHz. Prism was used to plot data and for statistical analysis.

## Reagents

Plasmin was purchased from Calbiochem (cat # 527624-10U).
