# Corrigendum: Effect of the protease plasmin on *C. elegans* hyperactive DEG/ENaC channels MEC-4(d) and UNC-8(d)

**DOI:** 10.17912/micropub.biology.000414

**Published:** 2021-06-25

## Description

For Johnson, CK; Miller, DD; Bianchi, L (2021). Effect of the protease plasmin on *C. elegans* hyperactive DEG/ENaC channels MEC-4(d) and UNC-8(d). microPublication Biology. 10.17912/micropub.biology.000412.

The authors correct the following:

1. The name of the author David D. Miller should read David M. Miller III

2. The affiliations

^1^University of Miami^2^Vanderbilt University

are corrected to:

^1^Department Physiology and Biophysics, University of Miami Miller School of Medicine, Miami, Florida, USA.

^2^Department of Cell and Developmental Biology, and Neuroscience Program, Vanderbilt University, Nashville, TN, USA.

3. The allele names that were used as reference for the cDNA for *mec-4* and *unc-8* are as follows:

– MEC-4(d) is MEC-4(A713T), corresponding to allele *u231*

– UNC-8(d) is UNC-8(G387E), corresponding to allele *n491*

4. The reference for the SitePrediction website (https://www.dmbr.ugent.be/prx/bioit2-public/SitePrediction/) is added as follows:

Verspurten J, Gevaert K, Declercq W, Vandenabeele P. SitePredicting the cleavage of proteinase substrates. Trends Biochem Sci. 2009 Jul;34(7):319-23. doi: 10.1016/j.tibs.2009.04.001. Epub 2009 Jun 21. PMID: 19546006.

These corrections do not alter the conclusions or discussion of this microPublication.

